# Supplementation of multienzyme with acidifier alleviates the antinutritional effects of a high soybean meal diet for nursery pigs

**DOI:** 10.3389/fvets.2025.1589827

**Published:** 2025-05-13

**Authors:** Ashir F. Atoo, Andres F. Bolivar-Sierra, Jorge Y. Perez-Palencia, Crystal L. Levesque, Rob Patterson, Hari B. Krishnan, Jinsu Hong

**Affiliations:** ^1^Department of Animal Science, South Dakota State University, Brookings, SD, United States; ^2^CBS Bio Platforms, Calgary, AB, Canada; ^3^USDA-ARS, University of Missouri, Columbia, MO, United States; ^4^Department of Animal Science, University of Minnesota, Saint Paul, MN, United States

**Keywords:** antinutritional factors, feed additives, growth performance, gut health, weaning pigs

## Abstract

**Introduction:**

The current study aimed to alleviate these negative impacts in diets containing high levels of SBM by supplementation of an enzyme combination solely or together with an acidifier.

**Methods:**

A total of 240 weaned pigs (average body weight of 5.9 kg), were allotted to 5 dietary treatments with 8 replicates in a randomized complete block design. Dietary treatments were low soybean meal (SBM) diet (17 and 20% in phase 1 and 2), medium SBM diet (22 and 25% in phase 1 and 2), high SBM diet (30% in and 35% in phase 1 and 2), high SBM diet with multienzyme supplementation, and high SBM diet with multienzyme and acidifier supplementation. All pigs were fed a common corn-SBM-based diet in phase 3. Growth performance was measured at the end of each phase along with blood collection. One pig per pen was euthanized to collect intestinal tissue for histomorphology and ileal digesta for the analysis of secretory IgA (sIgA).

**Results:**

Pigs fed high SBM diets with multienzyme or with multienzyme and acidifier tended to have a greater (*p* < 0.10) ADG compared to pigs fed High-SBM diet during phase 2 by 12.5%. During the 2nd week, pigs fed high SBM diets supplemented with multienzyme or multienzyme plus acidifier had less (χ^2^ < 0.05) diarrhea incidence than those fed high SBM diet. Dietary treatment did not affect the serum concentration of T4, IGF-1, IgA, and IgE. Pigs fed high SBM diet with multienzyme and acidifier supplementation had a lower (*p* < 0.05) concentration of sIgA in the ileal digesta on d 14 compared to high SBM diet, which was similar to low SBM diet. Except for Gly and Pro, pigs fed high SBM diets with enzyme and acidifier had improved apparent ileal digestibility of amino acids, which were higher (*p* < 0.05) than those fed high SBM and similar to those fed low SBM diets.

**Conclusion:**

Supplementation with multienzyme or multienzyme and acidifier in high SBM diets could reduce the negative impact of high SBM inclusion in nursery pig diets by improving amino acids digestibility and reducing allergenic response in nursery pigs.

## 1 Introduction

In swine diets, SBM is commonly used as the primary plant-protein source in the United States. Apart from its availability, SBM has a highly digestible amino acids (AA) profile, which helps supplement the limiting AA that are deficient in cereal grains and complement the AA profile of other common cereal grains like corn and wheat used in swine diets (1, 2). With the anticipated rise in soybean crushing capacity, the availability of SBM is expected to grow substantially, driven by the expanding demand for plant-based proteins, biofuels, and other industrial applications, leading to increased opportunities for its use in livestock diets (3). For this reason, it has been projected that the price of SBM may likely drop because of these expansions. Therefore, there is a need to explore ways of using more SBM in pig diets for profitable production and to maximize the nutrient value of SBM.

The presence of anti-nutritional factors (ANF) in SBM limits its availability of nutrients in weaning pigs. In newly weaned pigs, the inclusion of SBM in diets has been usually limited to 20% due to the potential for allergenic reactions, inflammatory responses, and digestive disturbances when included at levels exceeding 15 to 20% of SBM (4, 5). This is because of the presence of ANF such as trypsin inhibitors, soy protein antigens (such as glycinin, and *β*-conglycinin), lectins, phytate, non-starch polysaccharides (NSP), and oligosaccharides (6, 7). However older pigs can better tolerate the presence of these soybean meal based ANF due to better developed digestive systems, which in turn allows greater dietary inclusion of SBM.

One of the strategies to improve nutrient availability and ameliorate the effect of dietary ANF in SBM is the use of exogenous enzymes such as phytase, protease, and carbohydrase. Phytase is used in pig diets to hydrolyze phytate to inositol as well as inorganic minerals such as phosphorus and calcium, therefore making available phytate-bound phosphorus in cereal grains and oil seeds feedstuffs such as SBM thereby improving the digestibility of phosphorous, calcium, AA, and energy (8). Dietary protease has traditionally been used to hydrolyze peptide bonds in proteins and has been reported to improve the digestibility of dry matter (DM) and crude protein [CP; (9)]. While keratinase is a more specialized protease that has been reported to hydrolyze proteins with cystine disulfide bonds such as keratin, collagen, casein, and elastin (10) and also the cystine disulfide bonds in glycinin and *β*-conglycinin (11). Carbohydrases are a group of enzymes such as glucanase, mannanase, amylase, and xylanase that can hydrolyze complex sugars into simple sugars and are used to target NSP in plant-based feedstuff like SBM (12).

Weaning pigs, due to their limited ability to produce HCl, have high gastric pH, which reduces their ability to effectively digest proteins in their diets (13). Acidifiers can decrease the pH of the stomach, which helps in protein digestion by activating proteolytic enzyme activity (14). Several organic acids, including acetic acid, butyric acid, citric acid, formic acid, lactic acid, malic acid, and propionic acid, are commonly employed as acidifiers in pig diets (15).

Thus, it was hypothesized that supplementation of a multienzyme cocktail, including phytase, carbohydrase, protease, keratinase, and/or acidifier, would mitigate the antinutritional effects of SBM, ameliorating the growth performance and health of nursery pigs fed high SBM diets. Therefore, the objective of this study was to evaluate the effects of multienzyme and acidifier supplementation in high SBM diets on growth performance, diarrhea incidence, AA digestibility, intestinal histomorphology, blood profile, and secretory immunoglobulin A (sIgA) in ileal digesta.

## 2 Materials and methods

### 2.1 Animals and housing

Experimental animal procedures were reviewed and approved by the South Dakota State University Animal Care and Use Committee (#2306-051A). A total of 240 pigs [initial body weight (BW) of 5.9 ± 0.8 kg; PIC Line 42; Camborough and Compart Duroc] weaned at 18 d of age were obtained from Swine Education and Research Facility, South Dakota State University (SDSU; Brookings, SD, USA). The pigs were separated into two groups in two different locations/facilities; A group of 120 weaned pigs was housed in the on-campus swine facility in SDSU (Brookings, SD) and the other group of 120 weaned pigs was housed in the off-campus swine facility in the SDSU Southeast Research Farm (Beresford, SD, USA). They were weighed and allotted to 20 pens (6 pigs/pen) in each facility and balanced by BW and sex. Pens in the on-campus swine facility had a metal slatted floor (1.2 × 1.8 m) and pens in the off-campus swine facility had half slated-concrete floor (1.2 m × 4.9 m). Each pen was equipped with a cup drinker, a three-spaced dry feeder, and a heat lamp. Room temperatures were maintained at 30 ± 1°C during the first week. Thereafter, the room temperatures were maintained at 28 ± 1°C throughout the experiment.

### 2.2 Experimental diets

Experimental diets were low SBM diet (LSBM; SBM 17% in phase 1 and 20% in phase 2), medium SBM diet (MSBM; SBM 22% in phase 1 and 25% in phase 2), high SBM diet (HSBM; SBM 30% in phase 1 and 35% in phase 2), high SBM diet with multienzyme supplementation (HSBM+Enz; 100 ppm of phytase, 500 ppm of multi-carbohydrase, 250 ppm of protease, and 25 ppm of keratinase), and high SBM diet with multienzyme and acidifier (0.5%) supplementation (HSBM+Enz + Acid). The combination of the enzyme products was selected based on an *in vitro* digestion experiment using the methods described by Kim et al. (16) and evaluating the degradation rate for DM, CP, glycinin and beta-conglycinin present in SBM. There were 12 different enzyme combinations tested in the *in vitro* digestion study (Supplementary Table 1) and based on the degradation of crude protein and allergenic proteins (glycinin and *β*-conglycinin), the enzyme combination with phytase 100 ppm, carbohydrases 500 ppm, protease B 250 ppm, and keratinase 25 ppm was chosen for the feeding trial (Supplementary Table 2). The phytase contained 5,671 U of phytase per gram (567 FTU/kg of diet), the multi-carbohydrase complex contained enzyme activities for 2,939 U of xylanase (1,470 U/kg of diet), 323 U of *β*-glucanase (162 U/kg of diet), 1,751 U of cellulase (876 U/kg of diet), 13,001 U of protease (6,500 U/kg of diet), 815 U of invertase (408 U/kg of diet), 3,202 U of pectinase (1,601 U/kg of diet), and 26,961 U of amylase per gram (13,480 U/kg of diet). The protease contained 45,019 U of protease per gram (11,255 U/kg of diet) and keratinase contained 774,499 U of protease per gram (19,363 U/kg of diet). The acidifier used in the current study contained 30% formic acid, 7% citric acid, 12% acetic acid, and 10% lactic acid. The individual enzyme products were provided by CBS Bio Platform (Calgary, AB, Canada), and the acidifier (PiCid-PG4, pKa 3.13–4.76) was provided by Pathway Intermediates Limited (Chicago, IL, USA). Diets were formulated to meet or exceed the nutrient requirements of nursery pigs (1) and provided in mash form through 3 phases during the entire period. The 3-phase feeding program was: phase 1 (0 to 1 weeks), phase 2 (1 to 3 weeks), and phase 3 (3 to 6 weeks). In phase 3, all pigs received a common corn-SBM-based diet (32% SBM). Titanium oxide was added at 0.3% to the phase 2 diets to assess ileal digestibility within this period. The analyzed composition of SBM and ingredient composition of the experimental diets that were used in this study are presented in Tables 1, 2, respectively.

**Table 1:** Analyzed nutrient composition of soybean meal (As-is basis).

Item	Solvent-extracted soybean meal
Moisture, %	11.64
Crude protein, %	46.85
Crude fat, %	0.70
Crude fiber, %	3.69
Ash, %	6.00
Trypsin inhibitor activity, TUI/mg^1^	4.76
Soy glycinin, μg/mL	2,785
Soy *β*-conglycinin. ng/mL	3.06

1 Activity is expressed as trypsin units inhibited (TUI) per milligram of sample.

**Table 2:** Diet formulation and chemical composition in experimental diets^1^.

Ingredient	Phase 1			Phase 2			Phase 3
	LSBM	MSBM	HSBM	LSBM	MSBM	HSBM	Common
Corn	42.76	38.33	31.24	53.04	48.62	39.76	62.35
Soybean meal	17.00	22.00	30.00	20.00	25.00	35.00	32.00
Dried whey	25.00	25.00	25.00	15.00	15.00	15.00	0.00
Fish meal	4.00	4.00	4.00	2.00	2.00	2.00	0.00
Soy protein concentrate^2^	6.00	6.00	6.00	3.00	3.00	3.00	0.00
L-Lysine HCl	0.55	0.39	0.13	0.55	0.39	0.07	0.34
DL-Methionine	0.26	0.22	0.15	0.23	0.18	0.10	0.14
L- Threonine	0.21	0.15	0.05	0.22	0.16	0.04	0.16
L-Tryptophan	0.08	0.05	-	0.09	0.06	-	-
L-Valine	0.22	0.13	-	0.25	0.16	-	-
L-Arginine	0.35	0.22	-	0.40	0.27	-	-
L-Histidine	0.12	0.07	-	0.14	0.09	-	-
L-Phenylalanine	0.24	0.15	-	0.27	0.18	-	-
Soybean oil	0.85	1.02	1.28	1.63	1.80	2.12	1.78
Monocalcium phosphate	0.40	0.34	0.24	0.69	0.62	0.49	0.90
Limestone	1.06	1.03	1.00	1.17	1.16	1.14	1.20
Salt	0.29	0.29	0.28	0.46	0.44	0.41	0.60
Swine vitamin premix^3^	0.05	0.05	0.05	0.05	0.05	0.05	0.05
Swine mineral premix^4^	0.15	0.15	0.15	0.15	0.15	0.15	0.15
Zinc oxide	0.30	0.30	0.30	0.25	0.25	0.25	0.20
Swine larvicide	0.13	0.13	0.13	0.13	0.13	0.13	0.13
Titanium oxide	-	-	-	0.30	0.30	0.30	-
Calculated composition							
Metabolizable energy, kcal/kg	3,400	3,400	3,400	3,400	3,400	3,400	3,350
Crude protein, %	21.99	23.44	25.75	20.24	21.68	24.59	20.67
Crude fiber, %	1.61	1.67	1.78	1.79	1.86	2.00	2.22
SID Lys, %^5^	1.50	1.50	1.50	1.35	1.35	1.35	1.23
SID Arg:Lys	0.93	0.93	0.93	1.01	1.01	1.01	0.93
SID Met + Cys:Lys	0.55	0.55	0.55	0.55	0.55	0.55	0.55
SID Thr:Lys	0.59	0.59	0.59	0.59	0.59	0.59	0.59
SID Trp:Lys	0.21	0.21	0.21	0.21	0.21	0.21	0.18
SID Val:Lys	0.71	0.71	0.71	0.74	0.74	0.74	0.66
SID His:Lys	0.38	0.38	0.38	0.41	0.41	0.41	0.39
SID Phe:Lys	0.69	0.69	0.69	0.76	0.76	0.76	0.71
Calcium, %	0.85	0.85	0.85	0.80	0.80	0.80	0.70
STTD P, %^6^	0.45	0.45	0.45	0.40	0.40	0.40	0.33
Proximate composition							
Dry matter, %	90.55	90.72	90.85	89.40	89.61	89.79	87.77
Crude protein, %	21.76	22.97	25.84	21.79	21.91	25.86	20.78
Ether extract, %	2.22	2.35	2.52	2.50	2.74	2.98	2.63
Crude fiber, %	1.47	1.61	1.77	1.74	1.75	1.81	1.96
Ash, %	7.09	7.05	7.07	6.37	6.60	6.89	5.08
Trypsin inhibitor activity, TUI/mg^7^	0.80	1.04	1.30	0.98	1.18	1.56	1.49

1 LSBM: low SBM diet (17 and 20% in phases 1 and 2), MSBM: medium SBM diet (22 and 25% in phases 1 and 2), HSBM: high SBM diet (30 and 35% in phase 1 and 2).

2 HP300 (Hamlet Protein Inc, Findlay, OH).

3 Provided the following per kilogram of diet: 11,011 IU vitamin A, 1,652 IU vitamin D_3_, 55 IU vitamin E, 0.04 mg vitamin B_12_, 4.4 mg menadione, 9.9 mg riboflavin, 61 mg pantothenic acid, 55 mg niacin, 1.1 mg folic acid, 3.3 mg pyridoxine, 3.3 mg thiamine, and 0.2 mg biotin.

4 Provided the following per kilogram of diet: 165 mg Zn as ZnSO_4_, 23 mg Fe as FeSO_4_; 17 mg Cu as CuSO_4_, and 44 mg Mn as MnSO_4_.

5 SID: Standard ileal digestibility.

6 STTD: Standard total tract digestibility.

7 Analyzed by USDA-ARS [Columbia, MO; (18)].

### 2.3 Experimental design and procedure

The five experimental diets were allotted to the 40 pens (20 pens in each facility) with 6 pigs per pen in a randomized complete block design (*n* = 4 blocks in each facility; each comprised of 5 pens, with each pen representing a different treatment). Diets and fresh water were provided to pigs *ad libitum* during the entire period. Pigs were treated when they exhibited clinical signs of illness, and the treatment with pig identification was recorded throughout the experimental period.

Pig BW and pen feed disappearance were measured at the end of each feeding phase to calculate average daily gain (ADG), average daily feed intake (ADFI), and gain:feed ratio (G:F). To assess fecal consistency, pen fecal scores were assigned daily from Phase 1 to 2 and 3 times a week during Phase 3. One trained person assessed the fecal consistency in each pen. The classification scale had four descriptive categories, with score 1 being firm and shaped feces, score 2 being soft and shaped feces, score 3 being loose feces, and score 4 was for watery feces (17).

At the end of each feeding phase (days 7, 21, and 42), one pig (per pen) with BW that was close to the pen average BW was selected and blood samples were collected in serum vacutainer tubes (BD vacutainer; Becton Dickinson, Flanklin Lakes, NJ, USA) through the jugular vein of the pig. Serum was obtained by centrifuging at 1,872 × g for 15 min at 4°C. Thereafter, the serum samples were stored at −20°C until further analysis of thyroxine (T4), insulin-like growth factor-1 (IGF-1), immunoglobulin A (IgA) and immunoglobulin E (IgE).

On days 7 and 14, one pig (per pen) with BW close to the pen average BW was selected and euthanized using a captive bolt penetration. For gut histomorphology, intestinal tissues from 3 cm of jejunum (middle of small intestine) and ileum (70 cm from end of ileo-cecum junction) were cut and gently flushed with saline and collected in 50 mL conical tube with 10% neutral buffered formalin (Sigma-Aldrich, St. Louis, MO, USA) for later analysis. Ileal digesta sample was collected from the ileal segment of the small intestine, snap frozen in liquid nitrogen, and stored at −20°C freezer to determine the apparent ileal digestibility of CP and AA, and sIgA concentration.

### 2.4 Sample preparation and analyses

The SBM and experimental diets were ground to pass through a 0.75-mm screen using a centrifugal mill (model Zm200; Retsh GmbH, Haan, Germany). The ground SBM and diet samples were analyzed for DM, CP, ether extract (EE), crude ash, and trypsin inhibitor units. The samples were analyzed for DM by oven drying at 135°C for 2 h (method 930.15), CP by a combustion procedure (method 990.03), EE (method 2003.06), crude ash (method 942.05) as per AOAC (18), and trypsin inhibitor unit by a recent simplified method proposed by USDA-ARS researchers [Columbia, MO; (19)]. The SBM sample was analyzed for glycinin and *β*-conglycinin by enzyme-linked immunosorbent assay (ELISA) using commercially available kits (Glycinin: Soybean glycinin ELISA kit, Sunlong Biotech Co., LTD, Hangzhou, China, *β*-conglycinin: Soya Elisa kit II, Morinaga Institute of Biological Science, Inc. Yokohama, Japan).

Serum concentration of T4 was determined using Clinical Chemistry Auto-Analyzer System (Animal Disease Research & Diagnostic Laboratory, SDSU, Brookings, SD, USA). The ELISA was used to determine the serum concentration of IGF-1 (Porcine IGF-1 kit, Biomatik USA, LLC, Wimington, DE, USA), IgE (Porcine IgE kit, Biomatik USA, LLC, Wimington, DE, USA), and IgA (Porcine IgA kit; Bethyl Laboratories, Inc., Montgomery, TX, USA) according to the manufacturer’s guidelines.

The concentration of sIgA in ileal digesta was determined using ELISA according to the manufacturer’s guidelines (Porcine sIgA ELISA kit; Biomatik USA, LLC, Wilmington, DE, USA). The ileal digesta was freeze-dried for 7 days and analyzed for DM by oven drying at 135°C for 2 h (method 930.15), CP by a combustion procedure (method 990.03), and AA (method 982.30E) as per AOAC (18). The apparent ileal digestibility (AID) of AA was calculated using the index method according to the equation described by Kong and Adeola (20).

Intestinal tissues for histomorphology were sent to the Animal Disease Research and Diagnostic Laboratory at SDSU (Brookings, SD, USA) for staining with hematoxylin and eosin. The villous height (VH; from the top of the villi to the villous-crypt junction) and crypt depth (CD; from the villous-crypt junction to the base) were measured at 4 × magnification using a microscope (Micromaster®, Fisher Scientific, Waltham, MA, USA) equipped with a 0.55 × wifi camera eyepiece (MC500-W 3rd Gen., Meiji Techno Co. LTD., Saitama, Japan) and Micro-Capture software (Meiji Techno Co. LTD., Saitama, Japan) in 20 well-oriented villi and crypt columns. The villous height-to-crypt depth ratio (VH:CD) was calculated.

### 2.5 Statistical analysis

The UNIVARIATE procedure of SAS (SAS Inst., Inc., Cary, NC, USA) was used to confirm the homogeneity of variance. Data was subjected to ANOVA using the MIXED procedure of SAS in a randomized complete block design with the pen as the experimental unit and swine facility as the block. The model included treatment as the fixed effect and block as the random effect. Linear and quadratic contrasts for different spaced levels (SBM levels in phases 1 and 2) were performed to determine the effects of increasing inclusion of SBM in nursery diets. For the variable “fecal score,” data were subjected to the PROC FREQ procedure of SAS. When the Chi-square test was significant, *post hoc* pairwise comparisons between dietary treatments were conducted using Fisher’s exact test to assess specific treatment differences. Pair-wise comparisons using the Tukey post-hoc test were used to evaluate differences among treatment means when the overall ANOVA was *p* ≤ 0.10. To test the hypotheses, *p* < 0.05 was considered significant. If pertinent, trends (0.05 ≤ *p* ≤ 0.10) are also reported.

## 3 Results

### 3.1 Growth performance

The effect of multienzyme and acidifier supplementation on the growth performance of nursery pigs is presented in Table 3. Increasing levels of dietary SBM decreased (Linear, *p* < 0.05) BW on d 14. However, there was no effect (*p* > 0.05) of dietary treatment on BWs throughout the entire period. Dietary treatments tended to influence (*p* < 0.10) the ADG for 1–3 weeks, with pigs fed a higher SBM level diet showed lower ADG (Linear, *p* < 0.10) and pigs fed HSBM+Enz and those fed HSBM+ Enz + Acid tending to have greater (*p <* 0.10) ADG than those fed HSBM but similar to the pigs fed LSBM and MSBM. As the dietary SBM level increased, the ADFI of pigs for 1–3 weeks, 0–3 weeks, and 0–6 weeks were decreased (Linear, *p* < 0.05). Similar to the LSBM and MSMB, the HSBM+Enz + Acid and HSBM+Enz showed greater (*p* < 0.05) ADFI for 1–3 weeks and tended to have a greater (*p* < 0.10) overall ADFI when compared to the HSBM. The G:F was not affected (*p* > 0.05) by dietary treatments throughout the entire period.

**Table 3:** Effects of multienzyme supplementation and acidifier on growth performance of nursery pigs fed different levels of SBM.

Item	Dietary treatments^1^					SEM^2^	*p*-value^3^		
	LSBM	MSBM	HSBM	HSBM + Enz	HSBM + Enz+ Acid		Lin.	Quad.	Trt.
Body weight, kg									
Initial	5.94	5.96	5.94	5.99	5.99	0.150	0.999	0.957	0.998
Day 7	6.65	6.58	6.56	6.50	6.47	0.184	0.626	0.775	0.965
Day 14	8.40	7.91	7.67	8.22	8.14	0.229	0.046	0.430	0.219
Day 21	11.76	11.42	10.91	11.58	11.58	0.347	0.109	0.933	0.494
Day 42	26.11	24.33	23.96	25.70	25.89	0.791	0.110	0.347	0.214
Average daily gain, g/d									
0–1 weeks	102	78	76	85	84	12.1	0.376	0.528	0.590
1–3 weeks	370^x^	353^x^	316^y^	360^x^	361^x^	13.8	0.087	0.966	0.079
0–3 weeks	280	259	237	267	267	12.6	0.130	0.795	0.187
3–6 weeks	683	615	621	673	681	23.7	0.158	0.171	0.114
0–6 weeks	482^x^	437^y^	429^y^	470^x^	474^x^	16.6	0.113	0.321	0.100
Average daily feed intake, g/d									
0–1 weeks	132	119	115	119	118	9.0	0.436	0.713	0.714
1–3 weeks	541^a^	513^ab^	453^b^	491^ab^	536^a^	19.0	0.022	0.943	0.014
0–3 weeks	405^a^	381^ab^	340^b^	368^ab^	397^ab^	14.5	0.041	0.975	0.027
3–6 weeks	1,112	1,051	1,016	1,108	1,125	33.5	0.111	0.585	0.124
0–6 weeks	759^x^	716^xy^	678^y^	738^xy^	761^x^	22.4	0.034	0.651	0.072
Gain to feed ratio (G:F)									
0–1 weeks	0.73	0.71	0.62	0.70	0.67	0.057	0.234	0.584	0.732
1–3 weeks	0.68	0.69	0.71	0.73	0.67	0.019	0.316	0.986	0.200
0–3 weeks	0.69	0.67	0.70	0.73	0.67	0.019	0.625	0.516	0.269
3–6 weeks	0.62	0.59	0.61	0.61	0.61	0.009	0.983	0.167	0.205
0–6 weeks	0.64	0.61	0.63	0.64	0.62	0.010	0.969	0.215	0.261

^ab^ within a row, means without a common superscript differ significantly (*p* < 0.05).

^xy^ within a row, means without a common superscript show a tendency to differ (0.05 ≤ *p* ≤ 0.10).

1 LSBM: corn-SBM based diet with low SBM inclusion levels (17 and 20% in phase 1 and 2), MSBM: corn-SBM based diet with intermediate SBM inclusion levels (22 and 25% in phase 1 and 2), HSBM: corn-SBM based diet with high SBM inclusion levels (30% in and 35% in phase 1 and 2), HSBM+Enz: HSBM diet with multienzyme supplementation, and HSBM+Enz + Acid: HSBM diet with multienzyme and acidifier supplementation.

2 Standard error of the mean.

3 Lin., linear effect; Quad., quadratic effect; Trt., treatment effect.

### 3.2 Fecal score

**Figure 1:**
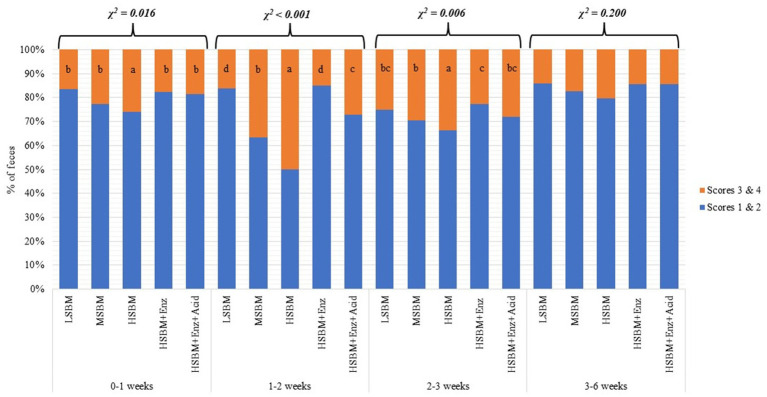
Effects of enzyme supplementation and acidifier on fecal consistency of weaned pigs fed different levels of SBM; The classification scale for fecal score had 2 descriptive categories with fecal score 1 and 2 were considered “no diarrhea,” while score 3 and 4 were considered “diarrhea.” ^abcd^ within a row, means without a common superscript differ significantly (*p* < 0.05). LSBM: corn-SBM based diet with low SBM inclusion levels (17 and 20% in phase 1 and 2), MSBM: corn-SBM based diet with intermediate SBM inclusion levels (22 and 25% in phase 1 and 2), HSBM: corn-SBM based diet with HSBM inclusion levels (30% in and 35% in phase 1 and 2), HSBM+Enz: HSBM diet with multienzyme supplementation, and HSBM+Enz + Acid: HSBM diet with multienzyme and acidifier supplementation.

Figure 1 showed that as the SBM inclusion level increased, the percentage of fecal scores 3 and 4 increased such that HSBM showed a higher (*χ*^2^ < 0.05) proportion of fecal scores 3 and 4 than other treatments in 0–1 weeks, 1–2 weeks, and 2–3 weeks. In 0–1 weeks, HSBM+Enz and HSBM+Enz + Acid treatments had no difference in diarrhea incidence compared with LSBM and MSBM treatments. In 1–2 weeks, HSBM+Enz and HSBM+Enz + Acid treatments had lower (*p* < 0.05) percentages of fecal scores 3 and 4 than MSBM and HSBM treatments. In addition, the HSBM+Enz treatment showed the least incidence of diarrhea, comparable to the LSBM treatment. During 3–6 weeks, when all pigs were fed a common diet, there was no difference (χ^2^ > 0.05) in fecal scores across dietary treatments.

### 3.3 Ileal amino acid digestibility

The effect of multienzyme and acidifier supplementation in high SBM diet on AID of AA in nursery pigs is presented in Table 4. The AID of Arg, His, Ile, Leu, Lys, Met, Phe, Thr, Trp, Val, Ala, Asp., Cys Glu, Ser, and Tyr decreased (*p* < 0.05) with increasing level of SBM inclusion (Linear, *p* < 0.05). However, HSBM+Enz + Acid treatment had higher (*p* < 0.05) AID of these AAs compared to HSBM treatment. The AID values of these AAs in pigs fed HSBM+Enz + Acid were comparable to the AID values of pigs fed LSBM.

**Table 4:** Effects of multienzyme supplementation and acidifier on apparent ileal digestibility of amino acids in nursery pigs fed different levels of SBM.

Item	Dietary treatments^1^					SEM^2^	*p*-value^3^		
	LSBM	MSBM	HSBM	HSBM+Enz	HSBM+Enz+ Acid		Lin.	Quad.	Trt.
Indispensable AA, %									
Arg	91.6^a^	83.6^ab^	72.8^b^	82.0^ab^	86.5^a^	4.10	0.012	0.829	0.041
His	88.4^a^	77.1^abc^	66.1^c^	74.6^bc^	81.0^ab^	4.66	0.008	0.610	0.030
Ile	86.8^a^	75.2^a^	54.0^b^	71.3^ab^	78.4^a^	6.25	0.005	0.992	0.014
Leu	86.4^a^	73.9^a^	55.6^b^	71.1^ab^	78.8^a^	6.14	0.006	0.869	0.020
Lys	91.4^a^	83.1^ab^	72.1^c^	80.0^bc^	85.3^ab^	3.58	0.003	0.774	0.011
Met	92.0^a^	84.5^ab^	73.9^b^	82.7^ab^	88.2^a^	3.57	0.007	0.859	0.016
Phe	91.4^a^	77.7^bc^	70.5^c^	74.7^bc^	85.7^ab^	4.45	0.011	0.333	0.016
Thr	86.5^a^	75.2^a^	59.0^b^	71.4^ab^	78.4^a^	5.26	0.004	0.838	0.015
Trp	91.9^a^	84.2^ab^	71.0^b^	79.8^ab^	86.4^a^	4.58	0.011	0.982	0.038
Val	88.0^a^	75.6^abc^	60.6^c^	71.9^bc^	81.2^ab^	5.39	0.007	0.750	0.016
Dispensable AA, %									
Ala	82.9^a^	69.9^a^	48.5^b^	65.0^ab^	74.5^a^	6.48	0.004	0.936	0.011
Asp	86.1^a^	74.5^a^	51.4^b^	69.4^ab^	74.8^a^	6.55	0.004	0.931	0.014
Cys	80.4^a^	66.4^ab^	45.4^b^	60.3^ab^	70.0^a^	7.07	0.006	0.889	0.023
Glu	88.1^a^	77.2^a^	57.2^b^	74.8^a^	77.5^a^	5.97	0.004	0.988	0.018
Gly	74.3^x^	62.8^x^	32.8^y^	55.4^xy^	61.8^x^	9.40	0.009	0.798	0.052
Pro	78.5	69.5	53.4	66.2	70.8	6.65	0.028	0.996	0.136
Ser	85.1^a^	74.0^a^	54.0^b^	69.8^ab^	75.7^a^	6.29	0.006	0.998	0.024
Tyr	87.9^a^	77.7^a^	61.2^b^	74.1^ab^	80.4^a^	5.22	0.005	0.923	0.018
Total AA, %	86.8^a^	75.8^a^	58.6^b^	72.0^ab^	78.5^a^	5.65	0.006	0.905	0.022
CP, %	84.1^a^	71.1^abc^	53.7^c^	66.1^bc^	75.8^ab^	6.08	0.005	0.800	0.019

^abc^ within a row, means without a common superscript differ significantly (*p* < 0.05).

^xy^ within a row, means without a common superscript show a tendency to differ (0.05 ≤ *p* ≤ 0.10).

1 LSBM: corn-SBM based diet with low SBM inclusion levels (17 and 20% in phase 1 and 2), MSBM: corn-SBM based diet with intermediate SBM inclusion levels (22 and 25% in phase 1 and 2), HSBM: corn-SBM based diet with high SBM inclusion levels (30% in and 35% in phase 1 and 2), HSBM+Enz: HSBM diet with multienzyme supplementation, and HSBM+Enz + Acid: HSBM diet with multienzyme and acidifier supplementation.

2 Standard error of the mean.

3 Lin., linear effect; Quad., quadratic effect; Trt., treatment effect.

### 3.4 Blood profiles

Table 5 showed the effect of multienzyme and acidifier supplementation on the blood profiles of nursery pigs. The serum concentrations of T4, IGF-1, IgA, and IgE for days 7, 21, and 42 were not affected (*p* > 0.05) by dietary treatments.

**Table 5:** Effects of multienzyme supplementation and acidifier on blood profiles of nursery pigs fed different levels of SBM.

Item	Dietary treatments^1^					SEM^2^	*p*-value^3^		
	LSBM	MSBM	HSBM	HSBM+Enz	HSBM+Enz+Acid		Lin.	Quad.	Trt.
T4, ug/dL									
Day 7	5.10	5.00	4.93	5.11	4.34	0.416	0.780	0.947	0.694
Day 21	4.26	3.93	4.28	4.95	4.29	0.312	0.877	0.457	0.253
Day 42	4.68	4.61	4.26	4.70	4.31	0.377	0.414	0.854	0.872
IGF-1, ng/mL									
Day 7	12.99	16.68	15.44	15.41	15.00	2.809	0.682	0.501	0.921
Day 21	14.71	10.76	11.53	10.54	11.70	1.754	0.303	0.221	0.469
Day 42	8.76	10.57	9.10	8.60	9.13	1.426	0.992	0.344	0.894
IgA, μg/mL									
Day 7	115.0	107.9	98.2	111.2	95.7	11.99	0.316	0.941	0.748
Day 21	669.1	612.9	695.2	548.4	483.4	78.62	0.756	0.551	0.326
Day 42	772.7	743.3	708.5	870.5	744.2	95.73	0.694	0.964	0.796
IgE, μg/mL									
Day 7	18.04	18.57	17.34	17.87	16.65	2.728	0.833	0.824	0.990
Day 21	27.37	25.61	25.63	27.68	26.75	1.670	0.533	0.577	0.854
Day 42	25.68	25.45	25.30	25.08	26.20	0.955	0.774	0.928	0.941

1 LSBM: corn-SBM based diet with low SBM inclusion levels (17 and 20% in phase 1 and 2), MSBM: corn-SBM based diet with intermediate SBM inclusion levels (22 and 25% in phase 1 and 2), HSBM: corn-SBM based diet with high SBM inclusion levels (30% in and 35% in phase 1 and 2), HSBM+Enz: HSBM diet with multienzyme supplementation, and HSBM+Enz + Acid: HSBM diet with multienzyme and acidifier supplementation.

2 Standard error of the mean.

3 Lin., linear effect; Quad., quadratic effect; Trt., treatment effect.

### 3.5 Gut histomorphology

The effect of multienzyme and acidifier supplementation on intestinal histomorphology of nursery pigs is presented in Table 6. Dietary treatments did not affect the histomorphology of jejunum and ileum for d 7 (*p* > 0.05). However, on d14, significant differences were observed, with pigs fed HSBM+Enz having a higher (*p* < 0.05) jejunal VH than those of pigs fed MSBM and HSBM+Enz + Acid. HSBM+Enz also had higher (*p* < 0.05) jejunal CD than those of pigs fed LSBM and MSBM, and HSBM+Enz + Acid. Furthermore, as SBM inclusion level increased, the ileal CD for d14 decreased (Linear, *p* < 0.05).

**Table 6:** Effects of multienzyme supplementation and acidifier on jejunal and ileal histomorphology of nursery pigs fed different levels of SBM.

Item	Dietary treatments^1^					SEM^2^	*p*-value^3^		
	LSBM	MSBM	HSBM	HSBM+Enz	HSBM+Enz +Acid		Lin.	Quad.	Trt.
Day 7									
Jejunal VH, μm	122.4	123.4	118.9	127.6	127.5	5.07	0.516	0.665	0.705
Jejunal CD, μm	77.4	78.1	75.9	78.8	72.2	3.30	0.733	0.784	0.643
Jejunal VH:CD	1.63	1.69	1.61	1.64	1.81	0.066	0.759	0.467	0.246
Ileal VH, μm	118.2	114.2	119.2	119.5	112.7	5.68	0.824	0.553	0.870
Ileal CD, μm	68.5	67.8	70.3	71.3	70.4	4.46	0.722	0.784	0.979
Ileal VH:CD	1.81	1.72	1.73	1.74	1.67	0.081	0.507	0.501	0.808
Day 14									
Jejunal VH, μm	171.7^ab^	153.7^b^	170.5^ab^	189.7^a^	159.4^b^	7.45	0.822	0.062	0.020
Jejunal CD, μm	96.3^b^	90.2^b^	99.9^ab^	109.2^a^	92.3^b^	4.39	0.413	0.220	0.037
Jejunal VH:CD	1.81	1.75	1.74	1.78	1.75	0.067	0.501	0.637	0.925
Ileal VH, μm	146.9	134.0	130.9	133.3	130.3	8.80	0.296	0.527	0.651
Ileal CD, μm	87.8	81.0	76.5	86.0	84.5	3.71	0.044	0.512	0.274
Ileal VH:CD	1.72	1.68	1.74	1.59	1.57	0.072	0.797	0.557	0.376

^ab^ within a row, means without a common superscript differ significantly (*p* < 0.05).

1 LSBM: corn-SBM based diet with low SBM inclusion levels (17 and 20% in phase 1 and 2), MSBM: corn-SBM based diet with intermediate SBM inclusion levels (22 and 25% in phase 1 and 2), HSBM: corn-SBM based diet with high SBM inclusion levels (30% in and 35% in phase 1 and 2), HSBM+Enz: HSBM diet with multienzyme supplementation, and HSBM+Enz + Acid: HSBM diet with multienzyme and acidifier supplementation.

2 Standard error of the mean.

3 Lin., linear effect; Quad., quadratic effect; Trt., treatment effect.

### 3.6 Secretory IgA in ileal digesta

Data on the effect of multienzyme and acidifier supplementation on sIgA concentration in the ileal digesta of nursery pigs is presented in Table 7. There was no difference in sIgA across dietary treatments on day 7. However, on day 14, the sIgA in the ileal digesta increased (Linear, *p* < 0.05) with increasing SBM inclusion levels, whereas pigs fed HSBM+Enz + Acid had a lower (*p* < 0.05) sIgA concentration compared to those fed HSBM and HSBM+Enz.

**Table 7:** Effects of multienzyme supplementation and acidifier on secretory IgA in ileal digesta of nursery pigs fed different levels of SBM.

Item	Dietary treatments^1^					SEM^2^	*p*-value^3^		
	LSBM	MSBM	HSBM	HSBM+Enz	HSBM+Enz+ Acid		Lin.	Quad.	Trt.
Secretory IgA, mg/mL									
Day 7	6.32	12.48	9.08	18.60	11.24	4.767	0.736	0.283	0.461
Day 14	22.73^b^	26.54^ab^	33.80^a^	32.56^a^	24.23^b^	2.791	0.006	0.965	0.027

^ab^ within a row, means without a common superscript differ significantly (*p* < 0.05).

1 LSBM: corn-SBM based diet with low SBM inclusion levels (17 and 20% in phase 1 and 2), MSBM: corn-SBM based diet with intermediate SBM inclusion levels (22 and 25% in phase 1 and 2), HSBM: corn-SBM based diet with high SBM inclusion levels (30% in and 35% in phase 1 and 2), HSBM+Enz: HSBM diet with multienzyme supplementation, and HSBM+Enz + Acid: HSBM diet with multienzyme and acidifier supplementation.

2 Standard error of the mean.

3 Lin., linear effect; Quad., quadratic effect; Trt., treatment effect.

## 4 Discussion

According to NRC (1), solvent-extracted SBM contains approximately 47.5% CP with a balanced AA profile high in Lys, Thr, and Trp, which are typically limiting in corn-based swine diets. Although SBM has an excellent AA profile, the presence of ANF such as lectins, trypsin inhibitors, and soy allergenic proteins such as glycinin and *β*-conglycinin have been reported to negatively affect the growth performance of nursery pigs (21, 22). The enzyme combination used in the present study was phytase, multi-carbohydrase (cellulase, pectinase, galactomannanase, and invertase), protease, and keratinase. As previously described, the combination and inclusion of the enzyme supplemented was determined in an *in vitro* study whereby the preparation used in the current study showed an improved capacity to degrade DM, CP, glycinin, and *β*-conglycinin present in SBM. Unfortunately, trypsin inhibitor activity in the *in vitro-*digested SBM was not detected using the USDA-ARS methodology. Based on this, it was hypothesized that this enzyme combination would alleviate the negative impacts of high SBM in the diets of nursery pigs.

The negative effects of high levels of SBM within nursery diets include interference in nutrient digestion (23), impairment of intestinal function (24), and increased incidence of diarrhea (4), all of which culminates in decreased growth performance of nursery pigs. In the current study, the high SBM diets were formulated with a high CP level and the same SID Lys, Met, Thr, Trp, and Val levels (Table 2). Increasing levels of CP, as associated with higher SBM inclusion, might have some negative consequences, such as causing a higher incidence of diarrhea, AA imbalance, and higher N excretion through deamination. This comes at a metabolic cost utilizing more energy that should have gone into growth and therefore reducing performance (25, 26). This may possibly explain the decreased growth performance and protein digestibility seen in the current study. The negative impact of increasing SBM inclusion levels in nursery pig diets has also been due to the inherent ANF, including oligosaccharides, NSP, allergenic proteins contained in SBM, which have been shown to negatively impact the pig growth by interfering with nutrient digestion and absorption (4, 6, 21). These negative effects of high SBM in nursery diets call for the careful formulation or adoption of strategies to mitigate these detrimental effects.

The enzyme combination used in the current study, either alone or with an acidifier, showed the potential to counteract the negative impact of dietary ANF of high SBM diets on the growth performance of nursery pigs. Previous studies have shown use of multienzymes or acidifier to favorably affect growth performance in weaned pigs. Omogbenigun et al. (27) reported an improvement in growth performance when a multienzyme cocktail, containing cellulase, pectinase, mannanase, invertase, xylanase, amylase, glucanase, protease, and phytase was supplemented in a corn-SBM diet containing low digestible ingredients. Previous studies have also reported the potential benefits of acidifiers on growth performance, as seen in the present study. Li et al. (28) reported an improvement in growth performance when a commercial acidifier was supplemented in the diets for nursery pigs. Also, a blend of citric, acetic, phosphoric, propionic, and lactic acids has been reported to improve growth performance of nursery pigs fed a corn-SBM diet (29). However, Li et al. (30) did not observe any significant effects of organic acid and enzyme combination on the growth performance of nursery pigs. Li et al. (30) used weaned pigs with an average BW of 10.5 kg and 35 days of age, fed diets containing 22% SBM. The difference between Li et al. (30) and the current study might be attributed to the different SBM inclusion levels, which could influence the negative impacts of ANF on young piglets. The results from the present study show that the enzyme blend, either alone or in combination with an acidifier, was effective in restoring the growth performance of nursery pigs fed high-SBM diets to levels comparable to those fed low-SBM diets. There was no additional benefit of the acidifier in terms of growth performance. Thus, the improvement in growth performance likely suggests that the enzyme combination used was effective in degrading the ANF known to negatively impact growth, and to a lesser extent, could be attributed to improved protein digestion, as indicated by the result for AID of AA.

The increase in diarrhea incidence with increasing levels of dietary SBM observed in the present study agreed with Zuo et al. (9), who reported a significant increase in diarrhea incidence when weaned pigs were fed high SBM (30%) diets. The increase in diarrhea incidence as the pigs were fed a high SBM diet is possibly due to lower digestion of CP and AA, leading to greater amounts of undigested enteric protein passing into the large intestine, which can cause digestive disturbances, which then manifest as diarrhea (31). The increase in AID of AA and CP as a result of the enzymes and acidifier used in the present study partly explained the reduced incidence of diarrhea observed in the pigs fed high SBM diets. Yu et al. (31) have reported similar results, showing reduced diarrhea in piglets fed diets supplemented with an exogenous protease preparation. The authors discussed that this effect was likely due to the increased protein digestibility, which decreases the accumulation of undigested protein in the hindgut. High amounts of undigested proteins in the hindgut promote the growth of pathogenic bacteria, which increases the incidence of diarrhea in weaning pigs (32, 33). The increased incidence of diarrhea in the present study may have partly contributed to the decrease in ADG and ADFI in pigs with increasing levels of dietary SBM, as diarrhea in weaning pigs has been reported to impair growth performance (34). In addition, the reduced incidence of diarrhea could partly be attributed to protease supplementation, as Zuo et al. (9) observed that dietary protease decreased the incidence of diarrhea in weaned pigs fed a high SBM (30%) diet without whey protein and fish meal. However, the enzyme cocktail used in the current study contained activities of various carbohydrases, protease, phytase, and other enzymes. Therefore, further studies are needed to determine which specific enzyme activity or other aspects of the dietary exogenous enzyme effects contribute to the reduced incidence of diarrhea in weaned pigs fed high-SBM diet.

There is a drastic decrease in the digestive enzyme activity in the stomach and pancreatic tissue immediately after weaning (35), implying that newly weaned pigs may not be able to digest the nutrients, specifically protein and AA. The result of the AID of AA in the present study showed the negative impact of increasing SBM levels in nursery diets on AA digestibility and how supplementing the high SBM diets with enzymes with or without dietary acidification affects the AID of AA and CP. Therefore, the result from this study showed the necessity of supplementing enzymes to increase the enzyme activity and supplementing acidifier to improve the endogenous enzymes efficacy (33, 35) to efficiently digest nutrients in newly weaned pigs fed high inclusion levels of SBM. The improvements in AID of CP and AA observed in the pigs fed high SBM diets with multienzyme plus acidifier has been attributed to the individual enzymes and acidifier used. The use of organic acids has been known to decrease the stomach pH which favors protein digestion and slows down the passage rate of feed through the gastrointestinal tract. The decrease in stomach pH improves protein and AA digestion by increasing the activation of proteolytic enzymes such as pepsin (32, 33). Results from the current study showed that the combination of multienzyme and acidifier improved the AID of AA more than multienzyme supplementation alone. This implies an added advantage of acidification, as it has been suggested that acidifiers may enhance the efficacy of exogenous or endogenous enzymes by providing a better pH range for enzymes involved in protein and AA digestion (33).

Protease increases the hydrolysis of dietary proteins to peptides and free AA, which are more readily absorbed in the small intestine. Furthermore, proteases have been reported to improve protein digestibility by neutralizing protease inhibitors in SBM, which can potentially inactivate trypsin and chymotrypsin (36), and by stimulating the synthesis of digestive enzymes, which can result in improved growth performance in weaned pigs (9). Also, keratinase has been reported to increase the AID of AA in nursery pigs fed a corn-SBM diet (37). Previous studies have demonstrated how different exogenous enzymes affect the digestibility of protein and AA. Carbohydrases have been reported to improve protein and AA digestibility by hydrolyzing plant cell wall components such as mannans, xylans, and *β*-glucans, thereby releasing AA trapped within the cell wall matrix (38, 39). SBM contains a high content of NSP (22.7%), including 14.5% cellulose and pectic polysaccharides, which negatively affect nutrient digestion and utilization in animals (40). Therefore, the selection of the carbohydrase blend containing cellulase and pectinase was appropriate for targeting cellulose, pectin, and soy oligosaccharides, and likely supported the improvement in the AA and CP digestibility for high SBM diet. Based on the above, it was evident that the enzyme combination used in this study worked synergistically to alleviate the negative impact of these ANF on AA digestibility.

Dietary treatment had no significant influence on serum parameters related to pig growth and immune or allergenic responses. Serum IGF-1, which is stimulated by growth hormone and secreted by liver, has been reported to have a positive correlation with feed efficiency and growth rate in pigs (41). Serum IgA and IgE, antibodies whose secretion is induced by feed antigens (22), showed no differences even with increasing levels of SBM. In the present study, it was expected that increasing levels of SBM in the diet would lead to decreased serum IGF-1 and T4 levels, reflecting a reduction in growth rate, as serum T4 has been reported to be positively correlated with serum IGF-1 (42). Furthermore, serum IgA and IgE levels, which are associated with antigenic or allergenic responses, were expected to increase in response to ANF from dietary SBM. However, no differences were found in these parameters. The reason for these observations could not be fully explained; however, it is possible that the difference in SBM levels was not sufficient to affect these serum biomarkers.

Soybean antigens promote the synthesis of sIgA by enteric mucous membrane as an immune response to dietary antigens in weaned pigs (43). It has been reported that sIgA is secreted as an immune defense mechanism against disruption of the intestinal barrier (44). The increasing levels of sIgA observed with higher dietary SBM inclusion could partly be attributed to the increased levels of dietary ANF in SBM, which may compromise the intestinal barrier and stimulate IgA secretion. Moreover, the decrease of sIgA concentration in the ileal digesta of pigs fed high SBM diets supplemented with multienzyme and acidifier may be explained by the mitigation of the detrimental effects of SBM-derived ANF, particularly attributable to the acidifier, resulting in less disruption of gut barrier function (45). Furthermore, the significant reduction in sIgA of ileal digesta in pigs fed high SBM diets with acidifier could be explained by the supplementation of organic acids promoting the degradation of allergenic proteins or allergens in conjunction with other enzyme activities, including carbohydrases and proteases, in the stomach and small intestine (46) or due to the antimicrobial properties of acidifiers (13). Even though sIgA was affected by dietary treatments, as noted earlier, there was no difference in serum IgA. The antibody IgA is associated with the immune function of the mucous membrane (in this case, the enteric mucous membrane). Therefore, the change in sIgA was probably not substantial enough to be detected in the blood or the response of the gut lumen to soy antigens was not pronounced enough to be noticeable in the serum. In other words, even with changes in IgA secreted in the intestinal mucosal membrane, the measurement location (serum) was likely not appropriate to detect the changes.

Results from the present study showed no differences in intestinal histomorphology with increasing levels of SBM or with enzymes plus acidifier supplementation compared with low, medium, and high SBM diets. The intestinal morphology was assessed on days 7 and 14 of post-weaning because more dramatic morphological changes in the small intestine have been reported to occur for at least 7 to 14 days post-weaning (45, 47). This was surprising, as the expectation was to find some differences in intestinal morphology, as dietary antigens from soy proteins have been reported to cause intestinal villous atrophy among other symptoms (48). Kim et al. (49) also found no differences in histomorphology when nursery pigs were fed a corn-SBM diet supplemented with a cocktail of enzymes containing protease, pectinase, xylanase, *α*-amylase, protease, and *β*-glucanase. Although there was no difference in jejunal VH:CD on day 14, the higher jejunal VH and CD in the HSBM+Enz treatment compared with MSBM and HSBM+Enz + Acid treatments remains unclear. Given the differences and the improvement in AID of AA among dietary treatments in the current study, it was expected that these differences would also be observed in the intestinal histomorphology. However, no improvement in intestinal histomorphology was observed. Intestinal histomorphology serves as an indirect index of digestibility in weaned pigs (50). Although the reason for the lack of differences in the intestinal histomorphology is not fully understood, it suggests that the increased AA digestibility observed in the current study is more reflective of the capacity of the enzymes to break down nutrients rather than an increase in the absorptive capacity on the small intestine.

## 5 Conclusion

Increasing the level of SBM inclusion in nursery pig diets negatively impacted growth performance, increased diarrhea incidence, and reduced amino acid digestibility, despite no significant changes observed in intestinal histomorphology or serum biochemical parameters. However, supplementation of high SBM diets with a combination of multienzymes and acidifier effectively mitigated these adverse effects. This additive strategy not only improved growth performance and reduced diarrhea but also enhanced amino acid digestibility to levels comparable with pigs fed low SBM diets. These findings suggest that using multienzymes and acidifiers is a practical and effective approach to overcome the challenges associated with high SBM inclusion, thereby enabling greater use of SBM in nursery pig diets without compromising pig health and performance.

## Data Availability

The raw data supporting the conclusions of this article will be made available by the authors, without undue reservation.
