# Supplementary material for: Supplementation of multienzyme with acidifier alleviates the antinutritional effects of a high soybean meal diet for nursery pigs
Source: Front Vet Sci. 2025 May 13;12:1589827. doi: 10.3389/fvets.2025.1589827 (PMC12106444; doi:10.3389/fvets.2025.1589827)
Supplement: Supplementary file 1 [file Table_1.docx]

**Supplementary Table 1**. In vitro digestion assay with 12 enzyme combinations

| Treatment | Phytase | Carbohydrases | Protease type 1 | Protease type 2 | Keratinase |
| --- | --- | --- | --- | --- | --- |
|  | Unit, ppm | | | | |
| 1 | 100 | - | - | - | - |
| 2 | 100 | - | 250 | - | - |
| 3 | 100 | - | - | 250 | - |
| 4 | 100 | - | 250 |  | 25 |
| 5 | 100 | - | - | 250 | 25 |
| 6 | 100 | - | 125 | 125 | 25 |
| 7 | 100 | 500 | - | - | - |
| 8 | 100 | 500 | 250 | - | - |
| 9 | 100 | 500 | - | 250 | - |
| 10 | 100 | 500 | 250 | - | 25 |
| 11 | 100 | 500 | - | 250 | 25 |
| 12 | 100 | 500 | 125 | 125 | 25 |

**Supplementary Table 2.** In vitro degradation rate for SBM with 12 enzyme combinations

|  | In vitro degradation rate, % | | | |
| --- | --- | --- | --- | --- |
| Treatment^1^ | Dry matter | Crude protein | Glycinin | β-conglycinin |
| 1 | 84.5 | 65.9 | 78.9 | 48.0 |
| 2 | 84.6 | 68.0 | 77.5 | 49.6 |
| 3 | 84.5 | 64.7 | 75.7 | 51.5 |
| 4 | 85.0 | 71.0 | 76.6 | 48.9 |
| 5 | 84.7 | 67.9 | 84.8 | 49.4 |
| 6 | 84.4 | 66.0 | 81.4 | 50.4 |
| 7 | 84.9 | 645 | 88.1 | 48.7 |
| 8 | 85.3 | 64.7 | 88.9 | 50.5 |
| 9 | 85.5 | 68.8 | 87.3 | 52.5 |
| 10 | 85.3 | 67.6 | 89.7 | 48.7 |
| 11^*^ | 85.6 | 73.8 | 88.2 | 50.8 |
| 12 | 85.6 | 69.8 | 88.1 | 51.5 |

^1^Treatment is described in supplementary Table 1. *Based on the in vitro digestibility (%), treatment 11 was chosen as the best enzyme combination, which was then used in animal study.
